# Hepatic metastasis from a meningeal hemangiopericytoma: A case report

**DOI:** 10.1097/MD.0000000000021605

**Published:** 2020-07-31

**Authors:** Aihemaiti Hasimu, Qiang Fu, Hui Wang, Qing-Jiu Zhou, Shao-Shan Li, Dangmu-Jiafu Geng, Chen Liu, Bo Liu

**Affiliations:** a Department of Neurosurgery; b Department of Pathology, First Affiliated Hospital of Xinjiang Medical University, Xinjiang, PR of China.

**Keywords:** meningeal hemangiopericytoma, hepatic metastasis, meningioma

## Abstract

**Introduction:**

A meningeal hemangiopericytoma (MHPC) is an aggressive tumor characterized by a high rate of local recurrence and late distant metastasis. The objective of this study was to share our experience with the treatment of a MHPC and how to distinguish this tumor from a meningioma.

**Patient concerns:**

A 62-year-old woman presented with symptoms of hypomnesia, hyperopia, and double vision for 1 month. Complete tumor excision was performed 6 years before. A biopsy sample was diagnosed as an atypical meningioma.

**Diagnosis:**

MHPC with late delayed hepatic metastasis.

**Intervention:**

Hepatic resection was performed initially, followed by secondary neurosurgery for complete excision of the bilateral frontal lesion 1 month later.

**Outcome:**

Based on the tumor pathology and consensus of oncologic surgeons, radiation therapy was initiated. Adjuvant therapy was well-tolerated and the patient remained recurrence-free at 6 months after surgery.

**Conclusion:**

Here, we report a case of local brain tumor recurrence and multiple hepatic metastases from a MHPC. Craniotomy combined with radical metastasectomy may be useful in such cases. Detailed immunohistochemical staining is helpful to distinguish a MHPC from a meningioma. Long-term follow-up is recommended.

## 1 Introduction

Hemangiopericytomas (HPCs) are rare vascular tumours arising from Zimmerman's pericytes, frequently located in the musculoskeletal system and the skin. The common anatomic locations of primary HPCs are the extremities, axilla, pelvis, retroperitoneum, head, and neck, and the sites of metastasis include the lung, bone, liver, pancreas, breast, and kidney.^[1]^

Here, we report a case of a recurrent meningeal HPC that had metastasized to the liver and was treated by craniotomy combined with metastasectomy 6 years after complete removal of the primary lesion.

## 2 Case presentation

A 62-year-old women presented with symptoms of hypomnesis, hypopsia and double vision for 1 month. She was previously diagnosed with bilateral frontal lobe meningioma and underwent tumour excision at our medical centre 6 years before. Since then, the patient remained healthy and complained of no neurological symptoms. The tumour pathology was interpreted as an atypical grade II meningioma according to the World Health Organization (WHO) criteria. No adjuvant chemo-radiotherapy was performed.

On admission, her blood pressure, body temperature, and heart rate were within normal limits. A physical examination revealed no abnormalities. Laboratory studies revealed a decrease in white blood cell and platelet counts. In order to eliminate hematopathy, the patient underwent bone marrow puncture, which revealed that all blood cells were within normal ranges. An overview of the patient's laboratory studies is described in Table 1.

**Table 1:**
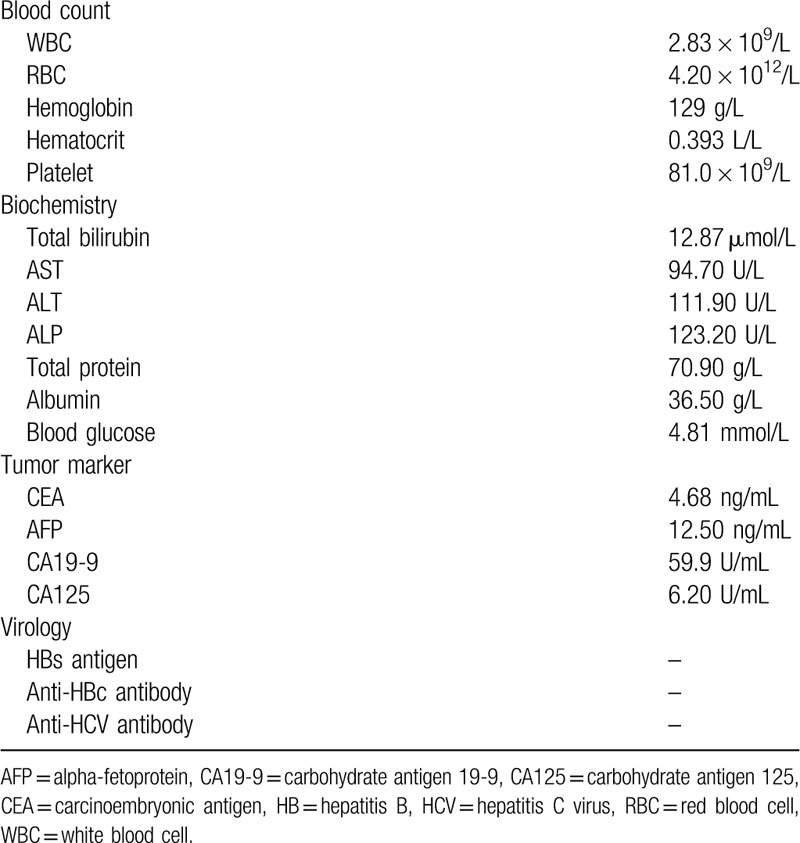
Patient laboratory findings on admission.

**Figure 1:**
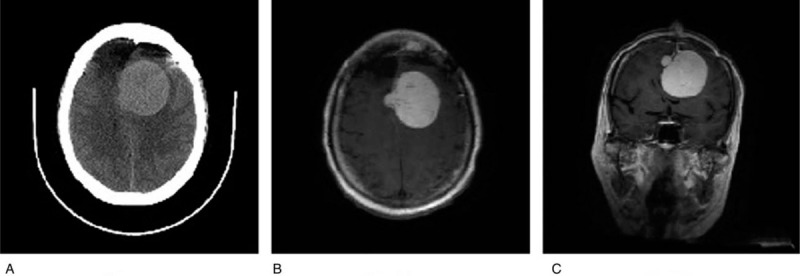
Pre-operative non-contrast computed tomography (CT) of the patient, axial view. (A) Pre-operative cranial magnetic resonance imaging, T_1_-weighted gadolinium contrast. Axial (B) and coronal (C) views of the bilateral frontal lesions.

Cranial magnetic resonance imaging (MRI) revealed 2 bilateral frontal lobe tumours. The larger 1 was located in the frontal lobe, measuring 4.49 × 4.60 cm at the largest dimensions, the smaller 1 in the right frontal lobe, measuring 1.11 × 1.00 cm. In addition, MRI showed a narrow dural attachment in both sides (Fig. 1).

**Figure 2:**
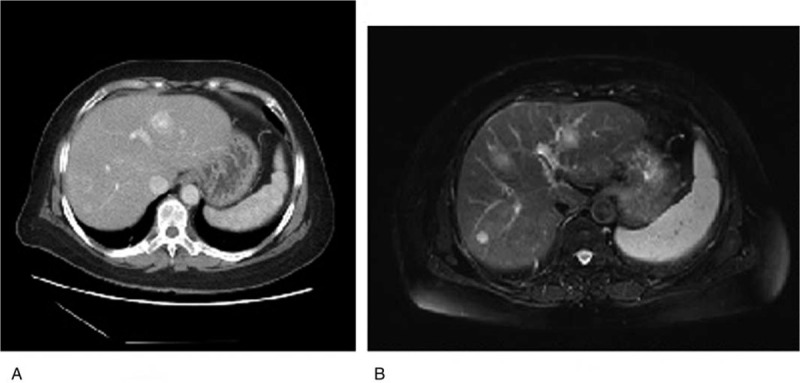
Computed tomography during arterial portography. (A) A hypervascular tumour with area of necrosis in both liver lobes, measuring 3.76 × 2.51 cm at the largest dimensions. (B) Pre-operative hepatic magnetic resonance imaging, T_1_-weighted gadolinium contrast.

Computed tomography (CT) during arterial portography showed that the larger hepatic tumour located in the right lobe measured 3.76 × 2.51 cm at the largest dimensions (Fig. 2A). Hepatic MRI (Fig. 2B) combined with laboratory studies confirmed the tumours were atypical hepatocellular carcinoma (HCC) intrahepatic metastasis. Full-body positron emission tomography/CT showed no extrahepatic metastases. In order to avoid tumour seeding, pre-operative percutaneous needle biopsy was not performed for histological diagnosis.

**Figure 3:**
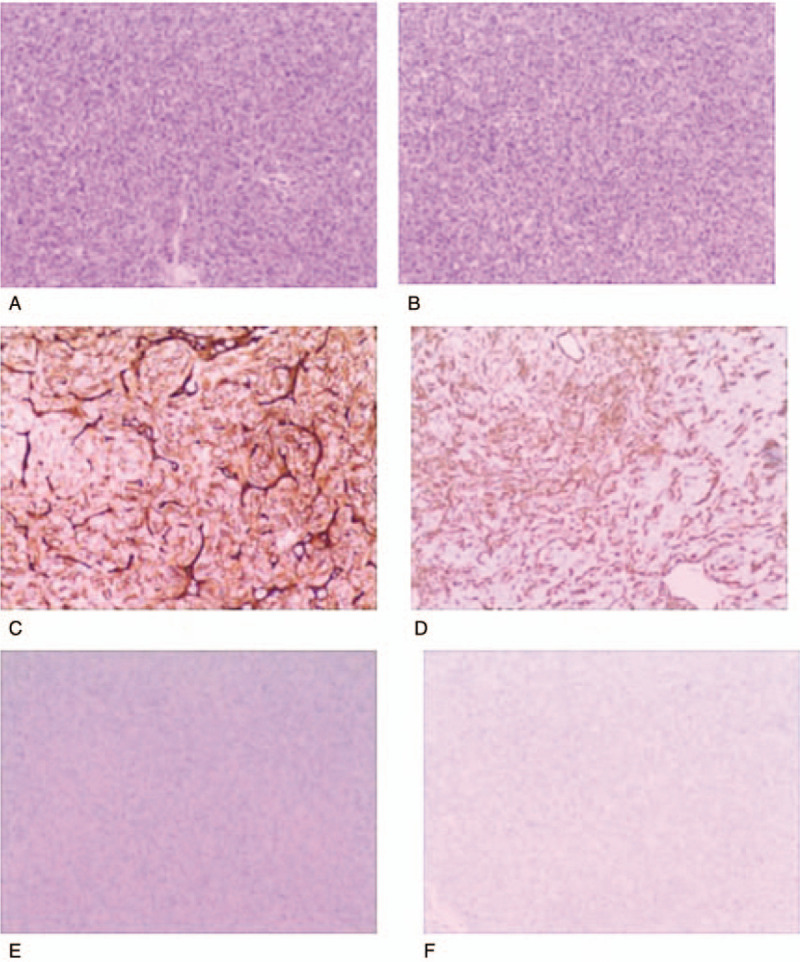
Cells are arranged around thin-walled vascular spaces, with a staghorn appearance, [hematoxylin and eosin (H and E stain, original magnification ×200] (A and B), confirming the hypervascularity and non-epithelial origin of the neoplasms, which exhibited a mild nuclear pleomorphism, low mitotic rate (<5 mitoses/10 HPF), and few areas of necrosis. Immunohistochemical analysis was positive for CD34 (C) and vimentin (D), but negative for epithelial membrane antigen (E) and glial fibrillary acidic protein (GFAP) (F). H&E = hematoxylin and eosin.

Under general anaesthesia, hepatic resection was performed and the patient had an uneventful postoperative recovery. Histopathological analysis revealed that the tumours consisted of relatively bland mesenchymal cells, with no unique characteristics, packed around an elaborate network of vessels. HPC WHO grade II was diagnosed by hematoxylin and eosin staining and immunohistological examination (Fig. 3).

**Figure 4:**
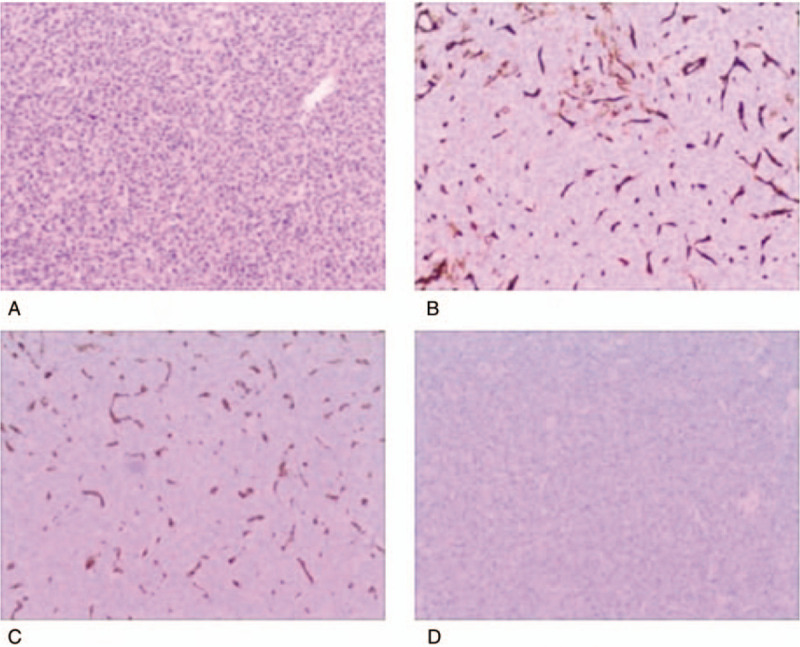
Photomicrographs of the recurrent tumour in the bilateral frontal region. (A) The tumour consisted of cells with oval nuclei arranged in a turbulent pattern surrounding slit-like ‘staghorn’ vascular channels (H and E stain, original magnification ×200.) (B) Immunohistochemical examination revealing positive staining for D34 via sporadic patterns in tumour cells. Immunohistochemical analysis was partly positive for vimentin (C) and negative for glial fibrillary acidic protein (GFAP) (D). H and E = hematoxylin and eosin.

**Figure 5:**
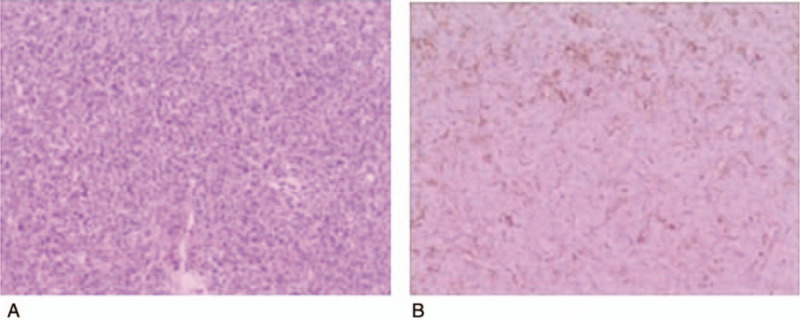
(A) Photomicrographs of the recurrent brain tumour in the frontal region at 6 years before. Densely packed elongated or polygonal neoplastic cells are arranged around branching staghorn-like vasculatures (H and E staining) from the brain original tumour 6 years ago. (B) Ki-67 mitotic index showing a median value of 10%. H and E = hematoxylin and eosin.

One month after the initial resection, the patient underwent a second neurosurgery to totally remove the bilateral frontal lesion. The lesion was WHO grade II–III, according to hematoxylin and eosin staining and immunohistological examination (Fig. 4). Rechecking of the pathological specimen of the original primary brain tumour from 6 years ago (Fig. 5) revealed that it was compatible with meningeal HPC. Therefore, the final diagnosis was meningeal HPC with late delayed hepatic metastasis.

One month later, the patient's postoperative Karnofsky Performance Score was 80. The patient underwent radiation therapy based on the tumour pathology and recommendations of oncologic surgeons. The patient tolerated the adjuvant therapy well and remains well and without recurrence 6 months after surgery.

## 3 Discussion

HPC is a highly vascularized neoplasm that presents in most patients with no pain or mass, while some develop neurological symptoms such as headache, paralysis and epilepsy.

There are several differentiating features of MHPC and meningioma. MHPC shows hyper-density on CT scans. MHPCs exhibit arterial phase enhancement and usually have well-defined borders on CT scans. MRI with gadolinium contrast reveals narrow dural attachment, irregular lobulated shape appearance and heterogeneous enhancement. Moreover, meningeal HPCs, which have a hyperplastic effect on adjacent bone, have been shown to exert an osteolytic effect. Presence of multilobulated tumour, iso-and hyperintensity on MRI and rare dural tail sign were the most important characteristics for differentiating MHPC from meningiomas. It's helpful to improve the diagnosis level through comparative analysis of the MRI and CT findings. Fluorodeoxyglucose positron emission tomography has been suggested to be helpful in revealing multiple distant metastases.^[2]^ It has been reported that HPC can be clearly distinguished from meningiomas because they have a larger peak at 3.56 ppm on magnetic resonance spectroscopy.^[3]^

We have reviewed the literature and found cases of late distant metastatic tumors from HPC similar to our case (Table 2).

**Table 2:**
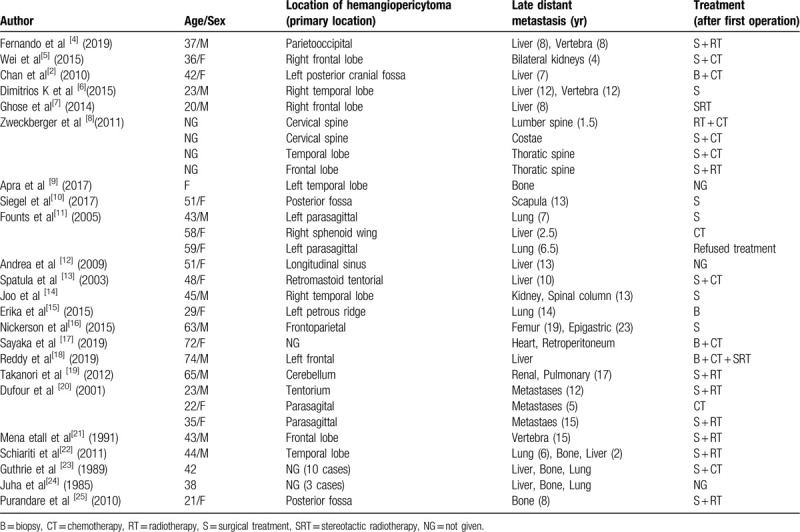
Reported cases of late distant metastasis from hemangiopericytoma.

HPCs are aggressive tumours that have a high rate of local recurrences and late and distant metastases. Extracranial metastases occur in 23% of cases and appear at a mean of 8 years after initial therapy, with the liver and bone as the most common sites of metastases.^[11]^ Extracranial metastases occur more than 5 years following craniotomy in most cases. A review of the literature retrieved nearly 35 cases of intracranial HPCs with hepatic metastasis. For the treatment of hepatic metastases, hepatectomy, radiotherapy, chemotherapy or transcatheter arterial chemoembolization alone or in combination are typically performed. According to the WHO classification, HPCs are graded as being differentiated (grade II) and anaplastic (grade III). However, irrespective of the histopathological classification, there is a significant consistency of data reporting that total tumour resection during the initial surgery is the most important factor for tumour control.^[26]^ Indeed, total surgical resection is regarded as the best treatment option to avoid recurrence or systemic metastases in patients with low-grade (grade II) HPCs.^[8]^

However, some authors recommend surgical excision and postoperative radiotherapy at a dose of 50 Gy or more as an initial treatment, as adjuvant radiotherapy results in significantly better local control than surgery alone.^[13]^ Furthermore, Rutkowski et al demonstrated that aggressive surgical intervention provides the best survival outcome regardless of tumour location and patient age, and that if radiation is included, the total radiation dose should be limited to <50 Gy.^[27]^

The use of postoperative stereotactic radiosurgery for tumours that are deeply located or cannot be removed with microsurgical techniques is associated with extended survival and maintenance of neurological function. Stereotactic radiosurgery, including Cyber-Knife radiosurgery and Gamma surgery, are effective for the treatment of meningeal HPCs and associated with a low risk of adverse effects.

Chemotherapy dose not result in any improvement in tumour control. However, a retrospective trial of 15 patients that investigated the effect of different chemotherapies demonstrated an overall survival rate of 14 months.^[28]^ Furthermore, anti-angiogenic agents have been proposed as potential promising therapeutic options for the treatment of HPCs.^[29]^

Microscopic examination is crucial for diagnosis of HPCs, which are composed of monomorphous spindle cells with moderate cellularity, arranged around staghorn blood vessels. HPCs and meningeal solitary fibrous tumours (SFTs) have significant similarities. Nevertheless, intracranial tumours are more cellular than HPCs or SFTs of soft tissues and contain fewer collagen bands. Additionally, meningeal HPCs demonstrate more mitoses, a higher Ki-67 index, and lower expression of CD34 and B-cell lymphoma 2 than HPCs and SFTs of soft tissues.^[12]^ Furthermore, careful histological and immunohistochemical examinations, including the recently discovered signal transducers and activators of transcription 6, are essential to achieve a correct diagnosis.^[30]^

## 4 Conclusions

Liver metastasis of meningeal HPCs follows a typical protracted clinical course. Salient imaging characteristics and histological features correlated with clinical history are helpful to achieve a correct diagnosis. Craniotomy combined with radical metastasectomy may be useful in these patients. Here, we report the longest duration between initial onset and the development of local recurrence and hepatic metastases of HPCs, suggesting that providing long-term follow-up is necessary after surgery. Primary staging examinations, such as CT of the chest, abdomen and spine, seem to be indispensable and are highly recommended.

## Acknowledgments

The authors would like to acknowledge Anaerguli Maimaiti for her help in the preparation of this report.

## Author contributions

**Aihemaiti Hasimu:** Participated in the sequence alignment and drafted the manuscript.

**Bo Liu:** Conceived the study and participated in the design, coordination and helped to draft the manuscript.

**Chen Liu:** Participated in the surgical consultation.

**Dangmu – Jiafu Geng:** Participated in the surgical consultation.

**Hui Wang:** Provided immunohistochemical images and analysis.

**Qiang Fu:** Participated in the study design.

**Qing – Jiu Zhou:** Performed the surgery.

**Shao-Shan Li:** Collected data and searched the Chinese and English literature.

All authors read and approved the final version of the manuscript
