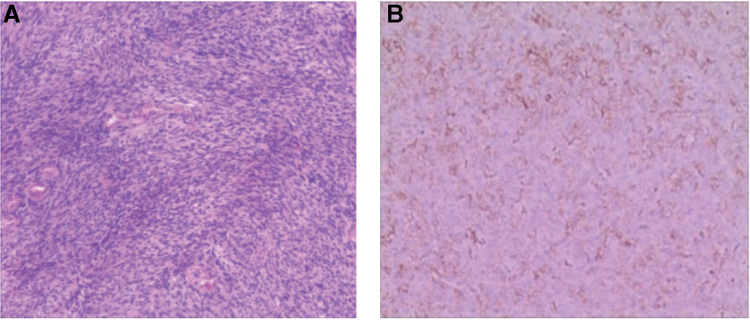# Hepatic metastasis from a meningeal Hemangiopericytoma: A case report: Erratum

**DOI:** 10.1097/MD.0000000000032983

**Published:** 2023-02-10

In the article, “Hepatic metastasis from a meningeal Hemangiopericytoma: A case report,”^[1]^ which published in Volume 99, Issue 31 of *Medicine*, the version of Figure 5A appearing in the article is incorrect. The correct figure is:

**Figure:**